# Advantages and disadvantages of channeling Gavi’s health system strengthening funds through health partners, a case study

**DOI:** 10.1371/journal.pone.0203647

**Published:** 2018-09-26

**Authors:** Charbel El Bcheraoui, Yodé Miangotar, Farah Daoud, Ellen Squire, Honoré Mimche

**Affiliations:** 1 Institute for Health Metrics and Evaluation, University of Washington, Seattle, WA, United States of America; 2 University of N’Djamena, N’Djamena, Chad; 3 Institut de Formation et de Recherche Démographiques, University of Yaoundé II, Yaoundé, Cameroon; Brandeis University, UNITED STATES

## Abstract

**Introduction:**

Following a period of interruption of Gavi’s funds for health system strengthening (HSS) in Cameroon and Chad, the two countries reprogramed their HSS grants. To implement the reprogrammed HSS, Chad committed to better management of the funds. Cameroon chose to channel the HSS funds through one of the health partners. This process is new to Gavi’s HSS grants, and little is known about its effectiveness or characteristics. We investigated the advantages and disadvantages of this process to inform the global health community about the added value of this solution.

**Materials and methods:**

We retrospectively evaluated Gavi’s HSS programs in Cameroon and Chad through a mixed methodology. To explore the pros and cons of channeling the funds through a health partner, we triangulated data from document review, key informant interviews (KIIs), field visits, and financial analysis of HSS expenditures in both countries.

**Results:**

Data triangulated from multiple sources showed that channeling HSS funds thorugh a health partner in Cameroon allowed compliance with budget, the development of a stronger accounting system at the Ministry of Health (MOH), and a rigid monitoring system. However, this mechanism delayed implementation by six months, accounted for 15% of the total cost, and created a tension around roles between MOH and the health partner. Achievement of program’s output indicators was average. In Chad, expenditures complied with budget as well. However, implementation was delayed longer causing a second reprogramming of the funds. While the program had fewer output indicators in Chad, these were minimally achieved.

**Discussion:**

To our knowledge, this is the first study of channeling Gavi HSS funds through a health partner. This new process contributed to a higher level of implementation, stronger monitoring, and strengthened accountability in Cameroon. Recipient countries of Gavi HSS grants who lack the financial management capacity can benefit from a similar process.

## Introduction

Gavi, the Vaccine Alliance (Gavi) aims at providing equal access to new and underused vaccines for children in the world’s poorest countries[1]. Gavi’s development assistance for health has grown by 538% since 2004, and in 2015, Gavi allocated $170 million– 10% of all its assistance–to health system strengthening (HSS) grants[2]. Following a 2004 report showing several examples of how poor health systems undermine the performance of immunization programs in developing countries, Gavi launched its first HSS window in 2005, and by 2008, 46 grants with cash disbursements for HSS totaling $230 million were already approved[3,4]. Gavi’s HSS grants were initially designed to increase vaccine coverage in recipient countries by strengthening the capacity of the health system to deliver immunization and other health services[5]. The Gavi Strategy 2016–20 includes HSS as part of the second Strategic Goal, which is to increase effectiveness and efficiency of immunization delivery as an integrated part of strengthened health systems[6].

Gavi’s HSS grants have evolved over the years, incorporating lessons learned from every country that has benefited from them. However, these grants encountered a common hurdle in many recipient countries[7]. HSS grants were often suspended for one to three years from the first disbursement, generally due to mismanagement of the funds. The suspension means an interruption of the grant’s activities, followed by reprogramming of the remaining funds. At the root of this situation is often the lack of local guidelines for HSS funds management[7]. This leaves room for a wide variety of interpretations around the management of these funds, often leading to the problematic situation described above.

Cameroon and Chad were among the early recipients of Gavi’s HSS grants. Cameroon started HSS implementation in late 2007[8] but the program was suspended in 2010. Following two years of suspension, Cameroon reprogrammed Gavi’s HSS funds in 2013 for 15 months[9]. Chad started HSS implementation in late 2008[10]. The program was scheduled to end in 2012 but was suspended in 2010 and reprogrammed first in 2012 and for a second time in 2013.

For the reprogrammed funds, Cameroon and Chad opted for two different strategies. Chad committed to better management of the funds. Cameroon chose to channel the HSS funds through one of the health partners, benefitting from the partner’s established financial management system. This process is new to Gavi’s HSS grants, and little is known about its effectiveness or characteristics. Previous evaluations of HSS grants focused on barriers and facilitators of program implementation and highlighted the minimal guidance from Gavi, the limited monitoring and reporting systems, and the confusion around the scope and goals of HSS support [5,11], but no study has evaluated the added value of managing Gavi’s HSS funds through a health partner.

We investigated the process and effect of channeling Gavi’s HSS funds through health partners to inform the global health community about the added value of this solution.

## Materials and methods

We retrospectively evaluated Gavi’s financial support for health system strengthening in Cameroon and Chad through a mixed methodology. To explore the pros and cons of channeling the funds through a health partner, we triangulated data from document review, key informant interviews (KIIs), field visits, and financial analysis of HSS expenditures in both countries.

### Ethics statement

This study received institutional review board (IRB) approval as a non-human subject research determination from the University of Washington.

### Document review

A document review was conducted at the beginning of the study by the principal investigator (CEB) to describe the HSS programs and their history in Cameroon and Chad, refine the research questions, identify key informants (KIs), and develop key informant interview (KII) topic guides[12,13]. Documents were selected as they pertain to Gavi HSS programs in Cameroon and Chad[14]. We searched for 1) documents relevant to HSS assessment, such as Gavi HSS country proposals and accompanying responses, HSS documentation provided by Gav, Gavi HSS policies and procedures, strategic plans such as annual plans of the Expanded Program on Immunization and national health plans, and 2) evaluations, either internal or external, that address relevance and achievement of results such as other country end-of-grant evaluations of Gavi’s HSS, annual progress reports, progress reports of the Millennium Development Goals, and academic evaluations of Gavi HSS grants. Most documents were publically available through Gavi’s website. Few documents, such as expenditure reports for the financial analysis were obtained from MOH in Cameroon or Chad. All documents obtained were reviewed for content and none was excluded. Information extracted pertained to critical dates such as proposals submission and funds disbursement, HSS planned, reprogrammed, and implemented activities, expenditures, key players and decision makers, and other topic relevant to the evaluation.

### Key informant interviews

A list of potential informants for key informant interviews (KIIs) was developed from the document review with input from Gavi Secretariat and Ministries of Health (MOH) in Cameroon and Chad. KIs were selected through a purposive sampling strategy and are detailed in Table 1. These were stakeholders from the Ministry of Health and their various directorates, the Expanded Program on Immunization, the World Health Organization (WHO), UNICEF, bilateral donors, and local partners involved in the Gavi HSSs, such as civil society organizations. Representatives from Gavi Secretariat and Gavi Independent Review Committee were also interviewed. In Cameroon, 11 central-level, seven regional-level, ten district-level, and 11 health partner KIs were interviewed. In Chad, fourteen central-level, three regional-level, nine district-level, and eight health partner KIs were interviewed. Four KIs from Gavi and Independent Review Committee were interviewed for Cameroon and five for Chad.

**Table 1:** Participants in Gavi HSS evaluations in Cameroon and Chad by category and method of data collection.

County	Study audience	Key informants Interviews	Field visits questionnaires
Cameroon	Gavi secretariat and IRC*	4	0
	Health partners	11	1
	MOH^∞^, central level	11	1
	MOH, regional level	7	7
	MOH, district level	10	10
	Health centers managers	0	105
	Total	43	124
Chad	Gavi secretariat and IRC	5	0
	Health partners	8	0
	MOH, central level	14	5
	MOH, regional level	3	3
	MOH, district level	9	9
	Health centers managers	0	40
	Total	39	57
Total		82	162

*IRC: Independent Review Committee;

^∞^MOH: Ministry of Health

Several topic guides were developed using the results of the document review. Questions were asked to help better identify barriers to the implementation of HSS funding, how they have evolved over time, and whether implementation was successful in spite of identified barriers. A general topic guide detailing the questions of the evaluations is available as appendix 1. Data collection was completed face to face during June–July 2015, and September–October 2015 in Chad and Cameroon, respectively. Researchers typed verbatim notes during KIIs. Each KII was conducted by two researchers and their notes were compared at the end of each day. Verbal consent was obtained at the beginning of each interview. Data from KIIs were explored through a thematic analysis technique. Where possible, findings from KIIs were triangulated with findings from reviewed documents for validation.

### Field visits

Field visits were conducted to verify implementation of HSS activities and evaluate the extent to which HSS objectives were met. Checklists of activities proposed in the original and reprogrammed HSS applications were transformed into questionnaires, by the principal investigator (CEB), to assess and verify the implementation of all activities. The questionnaires were reviewed for context-tailored language by the research teams from the University of N’Djamena in Chad, and the Institut de Formation et de Recherche Démographiques in Cameroon. These questionnaires were administered at various levels of the health system in Cameroon and Chad: central, regional, and peripheral. In Chad, where HSS was restricted to ten districts, we visited four HSS health districts and three control districts selected based on geographic and sociodemographic indicators similar to three of the four HSS districts, excluding the Capital’s district. In Cameroon, where HSS was a national program, we selected 11 health districts from seven regions representing the country’s geographic and sociodemographic variation. At the regional level, we met with the heads of the regional health delegations. At the peripheral level, in each sampled health district we met with the chief medical officer and the managers from a sample of health facilities. Data were collected using computer-assisted personal interviewing through offline data collection software. Table 1 represents study participants by category and method of data collection.

### Financial analysis

We analyzed HSS disbursements through a comparative auditing process. Each Gavi HSS proposal includes a budget for funds expenditure. Specifically, the financial analysis compared proposed expenditures in the HSS reprogramming proposal budget and actual expenditures. This comparison was conducted in order to assess adherence of expenditures to proposed and approved budgets. For the financial analysis in Cameroon, we used the proposed budget for the reprogrammed HSS proposal, as well as the financial report produced jointly by the MOH and the health partner[15,16]. For the financial analysis in Chad, we used the proposed budget for the first reprogrammed HSS proposal, as well as the financial expenditure report produced by the MOH[17,18]. Data from the financial analysis were triangulated with data from KIIs.

## Results

Following a two-year interruption of HSS funds, Gavi signed agreements with both Cameroon and Chad to reprogram the remaining funds that would be implemented through specific periods of time. In Chad, the management of reprogrammed HSS funds was allocated to the Planning Directorate, an existing entity at the Ministry of Health (MOH). In Cameroon, the country had two options: either develop new guidelines for the financial management of the HSS grant, or channel the funds through one of the health partners such as WHO, UNICEF, or similar organizations who are present in-country and provide the MOH with technical assistance. To avoid delays in creating guidelines for HSS grants management, Cameroon opted for the second option where a health partner would manage all HSS funds through a team appointed specifically for this task:

“*We chose to go to WHO because we had total confidence in WHO*, *and we didn’t give WHO only Gavi’s funds*. *We even gave our own money to facilitate the implementation*. *It was not an exception*, *it was already a practice from our own funds and activities*.”

In parallel, the team instituted at the partner organization would be mirrored by a local team at Cameroon’s MOH. The purpose was to transfer financial management skills to the MOH and build a more sustainable process for future grants. Cameroon chose a health partner with whom they had a long history of partnership, who has previously managed other health-related grants in Cameroon, and is trusted by the MOH.

### Advantages of channeling funds through the health partner

**Fig 1:**
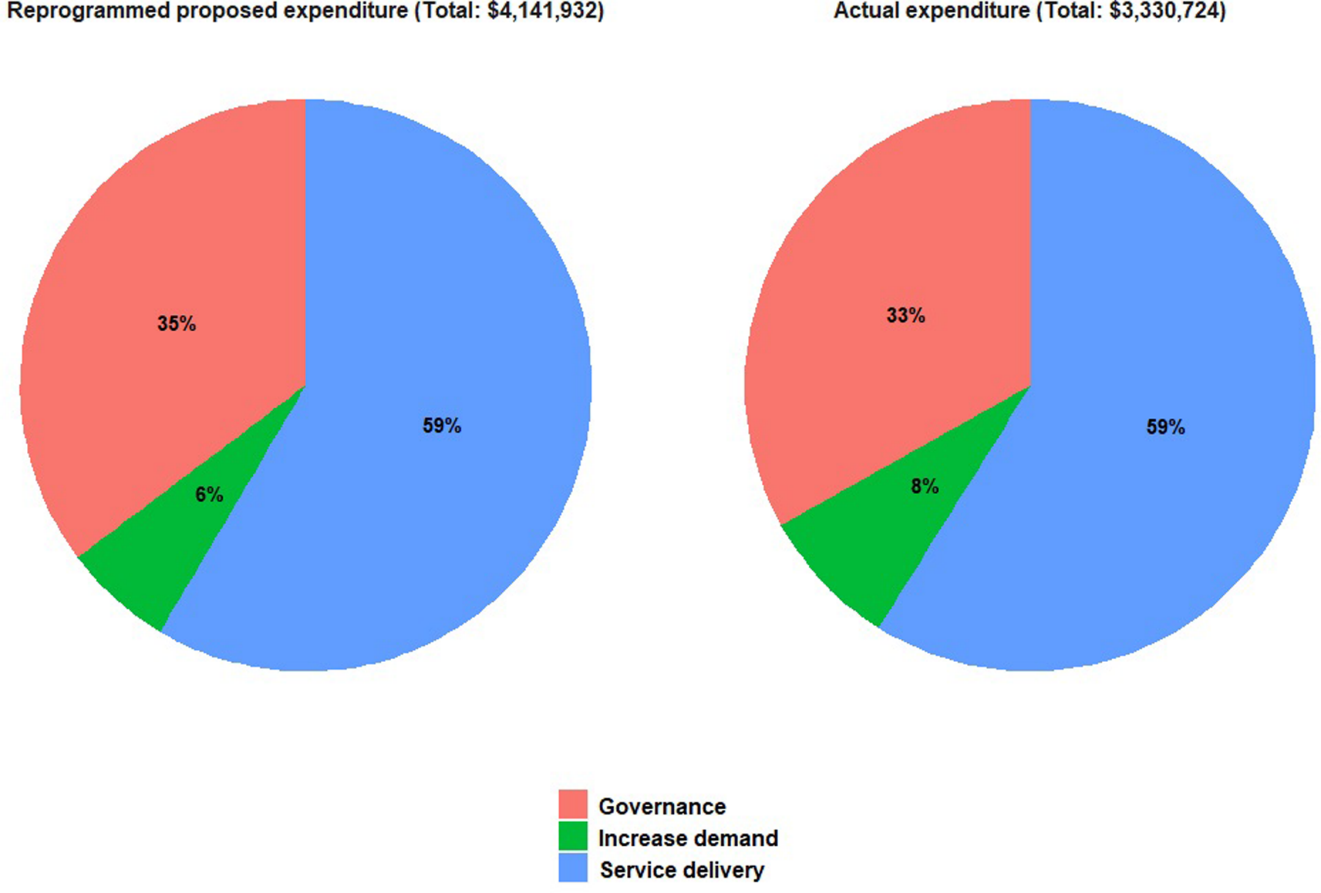
Proposed and actual expenditures of the reprogrammed HSS, Cameroon, 2014–2015.

In Cameroon, the health partner’s involvement in the management of HSS funds allowed compliance with the institution’s financial management procedures. All expenditures were tracked through a strong accounting system. This system requires users to document the way they spend the money, and a receipt should be submitted for each expenditure within a specific period of time before more funds can be disbursed. By the end of this evaluation, funds had been spent according to budget (Fig 1).

**Fig 2:**
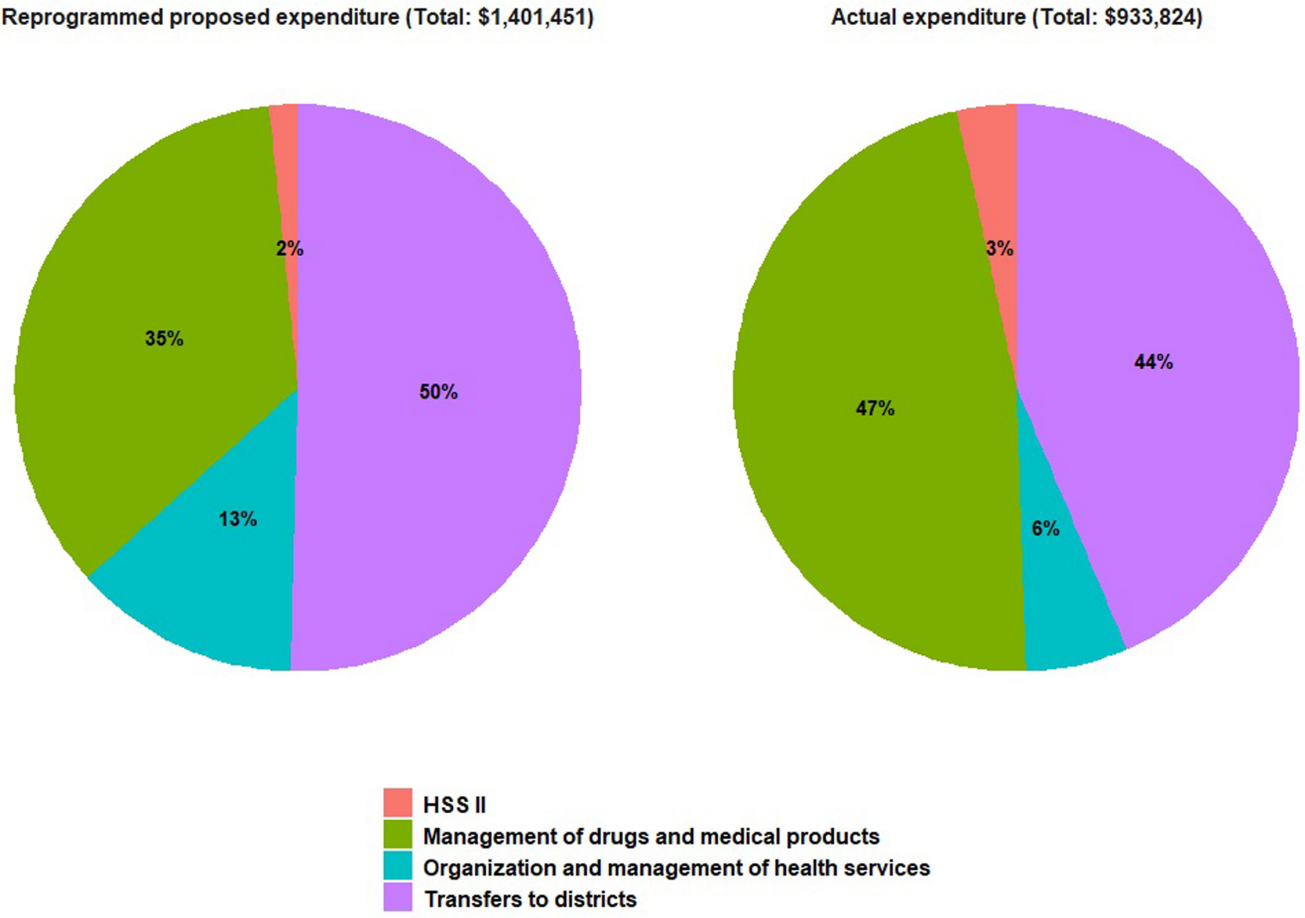
Proposed and actual expenditures of the reprogrammed HSS, Chad, 2013–2015.

In Chad, the Directorate of Planning managed HSS funds through the usual channels of the MOH. By the end of this evaluation, funds had been spent according to budget as well (Fig 2).

Second, due to the health partner’s accounting system, the MOH in Cameroon has evolved to the point where receipts are better managed and timelier:

“MinSanté has evolved to the point where receipts are better managed and timely, and a manual of financial management is being developed.”

The accounting system requires receipt for every expense before more funds can be disbursed. This has created a culture of accountability at the MOH, and a manual of financial management was being developed. Indeed, the embargo on HSS funds, described in the following section, only occurred once.

In Chad, such a system does not exist, and receipts are not always available for certain activities due to an archiving problem. This problem is even bigger at the peripheral level, where there is sometimes non-compliance with the program’s budget.

In Cameroon, the HSS team from the health partner also worked with the local team at the MOH and a team of civil society organizations to monitor the implementation of activities through monthly meetings and field visits, producing monthly bulletins that tracked activities implemented and their outputs, and the project’s progress. Apart from the monthly meetings of coordination attended by partners, as well as the weekly EPI, the two teams regularly conducted field missions to verify the implementation of the program and produce monthly reports documenting the implementation of activities and the progress of the project. They also conducted monitoring meetings with the regions every six months. Thus Gavi received a report almost every three months from the health partner. Also, as HSS activities are part of the plan of the EPI, they are followed at the same time as those of the EPI. At the central, regional and even HD level, one team or the other can directly monitor the implementation, while follow-up at the operational level is provided by a team representing civil society organizations. By the end of the evaluation, achievement of output indicators was average, with some indicators being almost met and some not met at all (Table 2). For instance, the country was well on track for the in strengthening the leadership, coordination, and governance of regional delegations and health districts, but still behind on strengthening the logistical support of the EPI at the operational level indicating that not all funds have been yet absorber, or not all activities have been yet implemented.

**Table 2:** Degree of achievement of the indicators of the reprogrammed HSS, Cameroon, 2015.

Objective	Indicators	Results (level)	Target
**Involve communities in the EPI in all 181 health districts**	% of discussions with at least one person trained in EPI*	73% (health district) 45% (health district)	100%
	% of health districts that have formed at least five of the women’s associations in EPI	55% (health district)	100%
	% of health districts who have signed agreements of partnership with at least one community organization	50% (public health center)	60%
		50% (private health center)	
**Strengthen the logistical support of the EPI at the operational level**	% of health facilities identified with solar refrigerator	2% (public health center)	100%
		0% (private health center)	
	% of health districts identified with service providers who have been trained in EPI	18% (health district)	100%
		100% (public health center)	
		90% (private health center)	
**Implement at least 50% of the supervision at all levels**	Supervisions by the EPI completion rate	100% (regional)	100%
	% of areas of health having at least a motorcycle	49% (public health center)	50%
		15% (private health center)	
**Strengthen the leadership, coordination, and governance in 100% of regional delegations and in at least 80% of the health districts**	% of regional delegations which hold coordination meetings	86% (regional)	60%
	% of EPI accountants trained in the use of management ANN_WANGECI software	71% (regional)	100%
**Improve the quality and completeness of the data of the EPI in at least 50% of the health areas**	% of the health district in which at least one Member is trained in data quality	89%	40%
	% of the teleconference equipment installed	100% (central)	100%
	Completeness of the EPI monthly report of activities in the health district	Data not collected	100%
	Timeliness of EPI monthly report of activities in the health district	Data not collected	70%

*EPI: E*xpanded Program on Immunization;*

In Chad, monitoring was less rigid, with almost all HSS tasks managed by the Director of Planning. HSS activities were monitored as part of the National Health Development Plan’s activities, and not separately as highlighted by one of our KI:

“*This is the annual review*, *like the meeting that we just had*, *where everyone came to present their results*, *and at the end there is a general report*. *All the indicators of the PNDS*”.

The technical committee who developed the proposal held meetings every month to discuss the status of activities. The committee included representatives from MOH, including the EPI and the Directorate of Reproductive Health and Vaccination, WHO, UNICEF, and non-governmental organizations. However, according to KI, monitoring was not done as planned in the reprogramming request. The annual progress report mandated by Gavi remained the only tool for official monitoring of the HSS program. This report is produced once a year and often lacks reliable data on indicators. In addition, the HSS program had fewer output indicators in Chad, and these were minimally achieved (Table 3).

**Table 3:** Degree of achievement of the indicators of the reprogrammed HSS, Chad, 2015.

Objectives	Indicators	Results from visited health centers		Target
		HSS Health centers (31)	Non-HSS health centers (9)	
**Strengthen the management and organization in ten priority health districts**	Number of health centers that have been visited at least six times over the previous year, with a checklist having been used during visits	29 received at least one visit during the last quarter	9 received at least one visit during the last quarter	100%
		Average number of visits: 3 (1–11)	Average number of visits: 2.5 (1–6)	
		27 confirmed that a checklist was used during the visit	9 confirmed that a checklist was used during the visit	
**Strengthen human resources in ten priority health districts**	Number of health centers that have qualified health professionals, with the number required, and present in the responsibility area at least 10 of the 12 months	- 31 have at least one health care provider	- 9 have at least one health care provider	80%
		31 were open with the appropriate personnel at least 10 months	9 were open with the appropriate personnel at least 10 months	
**Strengthen the management and supply of vaccines and essential medicines in ten priority health districts**	The average number of days of stock-out of the ten essential medicines in the health centers	- For each of the ten essential medicines, at least two centers had a day of stock out during the last quarter	- For seven of the ten essential medicines, at least one center had a day of stock out during the last quarter	< 3 days/quarter
		Average number of stock out days: 16.0 (2.5–55.0)	Average number of stock out days: 30.1 (20.0–45.0)	

**Note:** the results presented here reflect only the HSS funds reprogrammed first. This is due to the fact that by the end of the evaluation, less than 10% of the funds reprogrammed later were spent.

### Disadvantages of channeling funds through the health partner

The first hurdle encountered by the reprogrammed HSS in Cameroon was a delay in the disbursement of funds. However, once disbursed, the funds were not put to timely use, as the team that would manage HSS at the Ministry of Health was not hired for another two months. Moreover, midway through implementation the HSS team appointed at the MOH was replaced with a new team.

Second, and due to limited human resources capacity at the MOH, the disbursement of HSS funds was blocked for about four months by the health partner. During this period, the same health partner managed the finances of 14 vaccination campaigns that took place in 2014 in response to a large poliomyelitis outbreak. Given the MOH’s limited capacity, the receipts for the campaigns lagged by three months, causing the health partner’s financial system to be blocked, including HSS funds:

“*In order to grant access to funds for an activity*, *WHO requests evidence of the use of funds already allocated*. *With the outbreaks of polio in 2013 and 2014*, *almost 14 vaccination campaigns were conducted over a period of 13 months*. *With the lack of human resources*, *we were lagging by three months in campaign receipts at the end of 2013*. *This triggered an embargo in the financial system of WHO*, *which meant that any disbursement of funds*, *including those of HSS*, *were blocked until all supporting documents were received*.*”*

This situation took four months to be resolved, during which no HSS activities were implemented. By September of 2015, implementation was still ongoing in Cameroon. In retrospect, two extensions of ten and 12 months were requested for the reprogrammed HSS, and up until June 2015 only 80% of the reprogrammed funds had been spent.

Third, this process had higher program management costs: 7% of the total of HSS funds went directly to the expense of the health partner HSS team, and 10% of the rest were devoted to the management of the program, of which 70% were also devoted to the health partner. For equity reasons, the salaries of the HSS team at MOH were at the same level as those of the health partner, which is justifiable in terms of fairness but negatively affects the efficiency of funds.

Fourth, the health partner’s HSS team sometimes made decisions and implemented activities without referring to the MOH. This conflict was resolved through monthly meetings of the two teams as observed in meeting minutes, and reported by several KI on both ends. Nevertheless, this conflict of roles was perceived as an infringement on the country’s sovereignty. This negative perception of the role of the health partner was not limited to the MOH. It has been reported by several KI from different backgrounds, and was also shared by other health partners involved in HSS and other major public health programs. As mentioned by a high level KI:

“*In fact*, *WHO’s mission in the country is supporting the country*, *technical support which is something good in facilitating things*, *but in the context of this reprogramming*, *we sometimes had the impression that WHO had the good intention to do well but exceeded its powers of council*. *It meant that sometimes they took decisions and the country as expected to follow*. *At one point there was a leadership conflict*, *but I think that it was resolved through coordination meetings*.”

Naturally, most of these issues do not pertain to Chad, given that the country continued to manage the HSS funds through the MOH. However, HSS funds were reprogrammed twice in Chad–first in September 2012, and next in August 2013, at the same time as the funds from the first reprogramming were received. The funds from the second reprogramming were received in July 2014. Unfortunately, all transfers from the central level to the operational level were suspended by the Minister of Public Health, stopping almost any activity, in an attempt to better manage the MOH’s finances. Hence less than 10% of the activities planned for the second reprogramming had been implemented by the end of the evaluation. With this cycle of reprogramming and delays of funds, and the shortage in staff at the Directorate of Planning, the country could not absorb all of the funds by the end of 2015.

## Discussion

Our study showed mixed results around the benefits of channeling Gavi’s funds for HSS through health partners. Cameroon, in comparison to Chad, was able to achieve more in terms of HSS implementation and outputs. Activities were better monitored, and a culture of accountability was strengthened, if not born, at the MOH in Cameroon due to the rigidity of the health partner’s financial management system. When an actor is required to justify its performance to a forum who can question and judge this performance, and impose consequences, based on this judgement, on the actor, an accountability framework is created[19]. This framework has worked better in Cameroon where funds were interrupted twice than in Chad. In both Cameroon and Chad, Gavi interrupted the funds when expenditures were not well justified. However, when receipts were not provided on time to the partner’s accounting system, the HSS funds were blocked. This has enabled the MOH to take a stronger approach tracking and providing receipts for all expenditures of funds managed by the partner. However, this came at the expense of higher costs, delayed implementation, and tension around the role of the health partner.

Channeling Gavi’s HSS funds through a health partner ensured efficiency in expenditures in Cameroon. Indeed, funds were spent according to the planned budget. However, we observed a similar outcome in Chad, where funds were managed by a directorate of the MOH. Nevertheless, this efficiency is arguable as the implementation of the reprogrammed HSS in both countries took far longer than planned. The original plan was for implementation of the reprogrammed funds to be completed at the end of 2014 in both countries. In reality, by the end of 2015 neither Cameroon nor Chad had yet finished implementing all HSS activities.

More positively, accountability became a major focus at the MOH in Cameroon due to the requirements of the health partner’s financial management system. This was paralleled by a strong implementation monitoring strategy in Cameroon, which was missing in Chad. Focus on accountability was not the case for Chad, where receipts lacked for many expenditures. Cameroon was also able to achieve more than Chad on the level of processes and outputs. In Chad, less than 10% of the second reprogrammed activities were executed by the end of our evaluation, while in Cameroon, many activities were completely implemented. Hence, three major benefits resulted from channeling HSS funds through the health partner: the birth of a culture of accountability at the MOH, a strong monitoring strategy, and a higher volume of implemented activities.

Unfortunately, the benefits from channeling the funds through the health partner were paralleled by many caveats.

The first implication was reduced efficiency and effectiveness. The time spent before the mirror HSS team was hired within the MOH in Cameroon, as well as the embargo of the financial management system, caused serious delays in the implementation. One of the objectives of the MOH mirror HSS team was to build capacity for financial management within the MOH. The replacement of this team midway through implementation was a loss to this capacity-building effort, which had to be restarted with the new team. The root cause behind these obstacles was the shortage of human resources at the MOH in Cameroon. In Chad, the program mainly relied on its director to manage most activities, while in Cameroon, human resources shortage created a triple obstacle: the delay in hiring the MOH HSS team, the absorption of most human resources capacity at the MOH by a large poliomyelitis outbreak over the HSS theoretical implementation period, and the resulting suspension of funds and implementation for over four months. As Wyss et al point out, implementation cannot precede human resources growth in the scaling-up of health intervention[20]. Furthermore, human resources shortages have been recognized as a barrier to implementation of Gavi programs since the advent of Gavi itself[21].

On top of all this was the added cost resulting from the process of channeling the funds through the health partner. About 15% of the funds were spent on management, which meant less funds spent on HSS activities. In comparison, HSS management cost was minimal in Chad.

The main negative outcome in Cameroon was the tension created around the role of the health partner. The classic role of the health partner is to provide technical assistance to the country. By making decisions without consulting with the MOH, the health partner was perceived as trying to take over the government’s leadership role. This tension, which was observed during our field visit, and which affected all involved stakeholders, seemed typical to us for a situation in which a country is subsidized.

Channeling Gavi’s HSS funds through health partners can be a beneficial solution when the MOH of recipient countries of lack financial management capacity. As we saw in Cameroon, this process allowed for stronger implementation, monitoring, and accountability in HSS expenditures. However, a few caveats need to be considered if Gavi and HSS recipient countries would like to use this process.

First, the decision to channel funds through health partners should be totally left to the recipient county to make, and with clear and predefined terms. This will ensure the respect of the sovereignty of countries receiving any global assistance for health.

Second, prior to the beginning of a similar process, it is important to strengthen frameworks for permanent communication between health partners managing the funds and the recipient country, to prevent situations where confusion of roles can lead to tension and have an impact on implementation. Hanson et al corroborate the idea that communication breakdowns block implementation and scaling-up of priority health services in developing countries[22].

Third, countries with conditions similar to those of Cameroon and Chad should invest seriously in their archiving and accounting systems. In this analysis, several key documents pertaining to financial management were not available to consult in Chad. In Cameroon, the absence of a strong and simple accounting system caused a four-month delay in implementation. Kimaro and Nhampossa highlight that effective health information systems are necessary for producing useful information for decision-making and resource allocation within health interventions[23]. This includes both numerical data and critical documents. Similarly, Kihuba et al highlight that in order to have access to quality health services, a robust accounting infrastructure is needed[24]. In parallel, the health partner should have a separation of funds management if it is managing funds for more than one program in the recipient country. The four-month delay could have been averted if the health partner had managed each fund separately.

Fourth, in developing countries, many partners intervene in the health sector. The structures and the staff at MOH are not able to effectively follow or implement the activities of each partner. For instance, in Chad, the Directorate of Planning has probably tried to manage activities funded by other partners at the same time as HSS. This can go the other way around as well. In Cameroon, for example, the implication of the health partner in HSS could have decreased its attention toward other programs, or its own activities. Hence, we suggest that additional management costs be budgeted separately, on top of the cost of HSS activities, so that the cost of management does not reduce the grant’s efficiency.

Our study is subject to some limitations. First, the archiving problem in countries made it impossible to access some important documents, or to verify the existence of others. Second, a number of KI were not available or easily located, especially those who played important roles at the beginning of HSS, given that HSS in these two countries go back as early as 2006. Finally, recall bias was a limitation with some KI, as these programs started more than eight years ago. Nevertheless, our study has two major strengths that make up for the limitations above. First, it is based on a mixed methodology that used different sources of data to investigate and verify each of the concepts studied. The triangulation of data from different sources allowed us to provide more robust findings and conclusions. Second, the evaluation of these programs was a collaboration between interdisciplinary teams from external and local institutions in both countries. Each of the institutions brought its strengths and expertise in evaluation methodology to create a collectively deep body of knowledge.

To our knowledge, this is the first study to explore the characteristics of channeling Gavi’s HSS funds through an in-country health partner. Previously, discussion of the management of financial assistance for health has been limited to cost[25]. This did not include any assessment of the involvement of third parties and the value of their management of the funds. Our findings highlighted the added value of such a process in strengthening monitoring and accounting systems in the recipient countries, and in improving implementation and financial absorption capacity. On the other hand, these advantages were paralleled with several caveats, mainly a tension around roles between the MOH and the health partner.

Channeling Gavi HSS funds through a health partner contributed to a higher level of implementation, stronger monitoring, and strengthened accountability in Cameroon. Recipient countries of Gavi HSS grants who lack the financial management capacity can benefit from a similar process. However, lessons learned from the Cameroon experience should be taken into consideration in order to ensure the effectiveness of channeling the funds through a health partner. It will be crucial to evaluate future cases of similar mechanism. These evaluations will help build the evidence base for best practices around managing financial assistance for health.
